# Incident Cognitive Impairment in Nonobese Versus Obese Adults Aged 50 Years or Older With Metabolic Dysfunction‐Associated Steatotic Liver Disease: A Propensity Score–Matched Cohort Study

**DOI:** 10.1155/ijh/7550224

**Published:** 2026-08-26

**Authors:** Akram Alnounou, Mohammad Alabbas, Riddhi Machchhar, EzzEIDien A. Ibrahim, Mohammad Alali, Ahmad Alhusen, Syed-Mohammed Jafri, Basile Njei

**Affiliations:** ^1^ Department of Internal Medicine, Western Michigan University Homer Stryker M.D. School of Medicine, Kalamazoo, Michigan, USA, wmich.edu; ^2^ Department of Gastroenterology, Hepatology and Nutrition, Cleveland Clinic, Cleveland, Ohio, USA, clevelandclinic.org; ^3^ Department of Medicine, Saint Peter′s University Hospital/Rutgers Robert Wood Johnson Medical School, New Brunswick, New Jersey, USA, duhs.edu.pk; ^4^ Department of Internal Medicine, University of Missouri-Columbia, Columbia, Missouri, USA, missouri.edu; ^5^ Department of Internal Medicine, Henry Ford Macomb Hospital, Clinton Township, Michigan, USA, henryford.com; ^6^ Department of Internal Medicine, Advocate Illinois Masonic Medical Center, Chicago, Illinois, USA, advocatehealth.com; ^7^ Department of Gastroenterology and Hepatology, Henry Ford Health System, Detroit, Michigan, USA, henryford.com; ^8^ Section of Digestive Diseases, Yale School of Medicine, New Haven, Connecticut, USA, yale.edu

**Keywords:** body mass index, cognitive dysfunction, cohort studies, dementia, metabolic dysfunction-associated steatotic liver disease (MASLD)

## Abstract

**Aims:**

Nonobese metabolic dysfunction‐associated steatotic liver disease (MASLD) may carry disproportionate neurodegenerative risk, but whether body habitus modifies cognitive risk within MASLD remains unresolved. We compared cognitive impairment between nonobese and obese adults aged 50 years or older with MASLD.

**Methods and Results:**

Retrospective cohort study with 1:1 propensity score matching in the TriNetX Research Network, a federated electronic health record network. Adults with a first MASLD diagnosis (2016–2022) meeting a metabolic‐risk criterion were classified by index body mass index as nonobese (18.50–29.99 kg/m^2^) or obese (≥ 30.00 kg/m^2^); underweight patients were excluded. The primary outcome was a composite of incident cognitive impairment (mild cognitive impairment [MCI] through dementia) over Days 366–1825. Hazard ratios (HRs) were estimated with Kaplan–Meier and log‐rank methods; a negative control (acute appendicitis) and an *E*‐value assessed bias. Matching yielded 36,021 patients per group (mean age 64.3 years; 57% women; all standardized mean differences < 0.10). Nonobese MASLD was associated with a higher risk of the composite cognitive outcome (2.9% vs 2.6%; HR 1.126, 95% confidence interval [CI] 1.031–1.230; *p* = 0.008; *E*‐value 1.50), driven by MCI (HR 1.221, 95% CI 1.078–1.384; *p* = 0.002). Estimates were null for incident Alzheimer disease, all‐cause dementia, vascular dementia, all‐cause mortality, and the negative control.

**Conclusion:**

Among adults aged 50 years or older with MASLD, nonobese status was associated with a modestly higher risk of incident cognitive impairment. Prospective validation in imaging‐ or biomarker‐confirmed MASLD cohorts is warranted.

## 1. Introduction

Metabolic dysfunction‐associated steatotic liver disease (MASLD), the term adopted in 2023 to replace nonalcoholic fatty liver disease (NAFLD) and defined by hepatic steatosis together with at least one cardiometabolic risk factor, is among the most common chronic liver conditions worldwide and is increasingly viewed as a systemic disorder with consequences beyond the liver [1, 2]. MASLD affects an estimated 25% or more of adults globally [2]. It is not confined to patients with obesity: In a meta‐analysis of the global NAFLD population, 40.8% of affected individuals were nonobese, and 19.2% were lean [3]. Cognitive impairment and dementia are leading causes of disability in older adults. Among adults aged 65 years or older, the estimated prevalence is roughly 10% for dementia and 22% for mild cognitive impairment (MCI) [4], and MCI becomes more frequent with age and often progresses to dementia [5]. Hepatic steatosis has been linked to accelerated brain aging and smaller total cerebral brain volume, independent of cardiometabolic risk factors [6]. Identifying which patients with MASLD face the greatest cognitive risk therefore has a direct clinical value.

Multiple observational studies link MASLD to cognitive decline. A systematic review found that people with NAFLD performed worse in some, though not all, cognitive domains [7], and a nationwide cohort reported MASLD associated with higher incidence of Alzheimer disease (AD) and vascular dementia, with adjusted subdistribution hazard ratios (HRs) of 1.10 and 1.20 [8]. These effect sizes are modest and vary across cognitive domains and populations, which has left the clinical relevance uncertain [7, 8]. Body habitus complicates the picture further. In older adults, higher body mass index (BMI) is often associated with lower dementia risk, a pattern termed the obesity paradox that may stem from weight loss during the preclinical phase of neurodegeneration [9, 10]. Nonobese and lean MASLD is now regarded as a clinically distinct entity with its own prognosis [11, 12], and lean MASLD carries higher liver‐related mortality than nonlean disease [13]. Two recent cohorts suggest the brain may follow the same pattern: Lean MASLD conferred a greater risk of incident AD than overweight or obese MASLD in a Korean population [14], and nonobese MASLD showed higher AD‐specific mortality than obese MASLD over 30 years of follow‐up [15]. Both signals are open to reverse causation and residual confounding, the same forces invoked to explain the obesity paradox, and neither study directly compared nonobese with obese MASLD for incident cognitive diagnoses in a multi‐institutional electronic health record (EHR) population using rigorous matching. Whether body habitus modifies the risk of incident cognitive impairment within MASLD therefore remains unresolved.

To address this gap, we conducted a retrospective cohort study with propensity score matching in the TriNetX Research Network. We compared adults aged 50 years or older with code‐defined MASLD who were nonobese with those who were obese. Because the nonobese group also includes overweight individuals, this phenotype is broader and more clinically common than the lean phenotype emphasized in prior studies. The primary outcome was a composite of incident cognitive impairment spanning MCI through dementia. Drawing on evidence that lean and nonobese MASLD carry a disproportionate neurodegenerative risk [14, 15], we hypothesized that nonobese MASLD would be associated with a higher risk than obese disease. By isolating body habitus within an at‐risk MASLD population, the study is intended to inform cognitive risk stratification and to motivate prospective evaluation of body size as a determinant of brain health in metabolic liver disease.

## 2. Methods

### 2.1. Study Design and Data Source

We conducted a retrospective cohort study with propensity score matching using the TriNetX Research Network, a federated EHR network that provides access to de‐identified EHR data (demographics, diagnoses, procedures, medications, and laboratory values) across participating healthcare organizations. Queries were run on the TriNetX “Research” network; the primary analysis drew on 111 participating healthcare organizations. Eligible adults were aged 50 years or older with a first recorded diagnosis of MASLD, identified by International Classification of Diseases, Tenth Revision, Clinical Modification (ICD‐10‐CM) codes K76.0 or K75.81, first recorded between January 1, 2016, and December 31, 2022. Because histologic confirmation was not available for cohort definition, K75.81 was included only as part of the code‐defined MASLD case‐finding strategy and was not used to define a separate histologically confirmed steatohepatitis subgroup. Analyses were generated on the TriNetX Analytics platform on June 12, 2026. TriNetX provides aggregate data that are de‐identified in accordance with the de‐identification standard defined in 45 CFR §164.514(a), with the de‐identification process attested to by a qualified expert under 45 CFR §164.514(b)(1). The investigators had no access to identifiable private information, could not ascertain individual patient identities, and had no interaction or intervention with patients. Accordingly, this secondary analysis did not involve human subjects as defined under 45 CFR §46.102(e), and institutional review board review was not required.

### 2.2. Study Design Rationale

To isolate the contribution of body habitus within an at‐risk metabolic liver disease population, we compared two groups that all met identical MASLD eligibility criteria and a shared baseline metabolic‐risk criterion, differing only in BMI category. Comparability of measured baseline characteristics between groups was achieved through 1:1 propensity score matching across demographics, cardiometabolic and vascular disease, obstructive sleep apnea, chronic kidney disease, neuropsychiatric disease, smoking, frailty and weight‐loss markers, liver severity, and selected medications.

### 2.3. Study Cohort and Eligibility Criteria

We included patients aged 50 years or older at their first MASLD code who met a baseline metabolic‐risk criterion and who had at least one healthcare encounter in the year before the index date. The metabolic‐risk criterion required at least one of the following, recorded on or before the index date: Type 2 diabetes, prediabetes or other abnormal glucose, hypertension (including hypertensive heart and/or kidney disease, secondary hypertension, or hypertensive crisis), disorders of lipoprotein metabolism, or metabolic syndrome/insulin resistance. The index date was the date of the first qualifying MASLD diagnosis. BMI category was assigned from a BMI measurement within 1 year before or on the index date: Nonobese was defined as BMI 18.50–29.99 kg/m^2^ and obese as BMI ≥ 30.00 kg/m^2^. Patients who were underweight (BMI ≤ 18.49 kg/m^2^) were excluded. We applied the following baseline exclusions, assessed before or on the index date: prior cognitive disease or dementia, including prescription of a dementia medication (donepezil, rivastigmine, galantamine, or memantine); competing or alcohol‐related liver disease and alcohol‐related disorders; and hepatic decompensation, including hepatic encephalopathy, or palliative care. Detailed analysis specifications are provided in Methods S1, and full outcome code definitions are provided in Methods S2.

### 2.4. Exposure and Comparator Definitions

The exposure group comprised nonobese patients with MASLD (BMI 18.50–29.99 kg/m^2^), and the comparator group comprised obese patients with MASLD (BMI ≥ 30.00 kg/m^2^), with BMI ascertained within 1 year before or on the index date. Because the nonobese group includes overweight individuals (BMI 25.00–29.99 kg/m^2^), it is broader than the lean phenotype and more clinically common. All HRs compare nonobese versus obese MASLD.

### 2.5. Outcome Definitions

The primary outcome was a composite cognitive outcome defined by any of ICD‐10‐CM codes G30, F01, F02, F03, G31.83, G31.84, and F06.7. Secondary outcomes were the prespecified cognitive components and related outcomes: MCI/mild neurocognitive disorder (G31.84, F06.7), incident AD (G30, G30.0, G30.1, G30.8, G30.9), all‐cause dementia (G30, F01, F02, F03, G31.83), vascular dementia (F01), and all‐cause mortality (TriNetX Deceased indicator). For each outcome, events were counted from Day 366 through Day 1825 after the index date, and patients with that outcome at any time before the start of the analysis window were excluded from the analysis of that outcome. Full code‐defined terms and analysis windows are listed in Methods S2.

### 2.6. Negative Control Outcome

To evaluate the possibility that unmeasured confounding or other systematic bias could generate spurious associations, we prespecified acute appendicitis (ICD‐10‐CM K35) as a negative control outcome, because body habitus within MASLD was not expected to affect its development. A null association was anticipated if the design and matching adequately addressed bias.

### 2.7. Sensitivity and Additional Analyses

Two sensitivity analyses were performed (Methods S1, Table S1, and Figure S1). First, a longer follow‐up analysis restricted index dates to 2016–2020 and extended the outcome window to Days 366–3650 (1–10 years), testing whether estimates changed when earlier indexed patients had a longer available outcome window; this analysis used the same full clinical matching approach as the main analysis. Second, a simpler adjustment model matched only on age, sex, race/ethnicity, and tobacco use/history, testing whether the more extensive clinical matching materially attenuated estimates. Because TriNetX is updated over time, the main analysis was repeated using the same query date and network as the additional analyses. This provided a directly comparable reference for assessing each analytic modification while retaining the original analysis as the primary analysis. The repeat and additional analyses were conducted after completion of the original analysis. Exact query dates, network size, and full specifications are provided in Methods S3 and Table S5.

### 2.8. Follow‐Up

For each outcome, follow‐up spanned the prespecified fixed analysis window from Day 366 through Day 1825 after the index date (1 through 5 years) in the main analysis, and from Day 366 through Day 3650 (1 through 10 years) in the longer follow‐up analysis. In the time‐to‐event analyses, patients were censored after the last recorded fact in their record. For the same‐cohort follow‐up analysis, a cohort restricted to the 2016–2020 index period was matched once and evaluated over both outcome windows.

### 2.9. Missing Data

Cohort assignment required a BMI measurement within 1 year before or on the index date. Patients without a qualifying BMI measurement were not eligible for classification into either exposure group. Diagnosis‐, procedure‐, and medication‐defined variables were based on recorded TriNetX aggregate terms, and absence of a recorded term was treated as absence of recorded evidence for that condition or exposure. Variable‐level missing‐data counts for these code‐defined covariates were not available in the de‐identified aggregate output. For each outcome analysis, patients with the relevant outcome before the analysis window were excluded, and time‐to‐event analyses censored patients after the last recorded fact in their record. Medication variables represented recorded RxNorm ingredient concepts used as matching characteristics. The aggregate output did not establish active or concurrent use, dispensing, dose, duration, adherence, or indication. An individual could contribute to more than one medication row.

## 3. Statistical Analysis

To reduce baseline imbalances, we used 1:1 propensity score matching implemented on the TriNetX Analytics platform. In the primary analysis, matching covariates spanned demographics, cardiometabolic and vascular disease, obstructive sleep apnea, chronic kidney disease, neuropsychiatric disease, smoking, frailty and weight‐loss markers, liver severity, and selected medications (full matched‐covariate lists in Tables S2, S3, S4). Covariate balance was assessed using the standardized mean difference (SMD), with an absolute SMD < 0.10 indicating negligible imbalance. Continuous variables were summarized as mean (SD) and categorical variables as counts and percentages. Risks (cumulative incidence within the window) and risk differences were calculated for each outcome. Time‐to‐event outcomes were analyzed using Kaplan–Meier estimation with a daily time interval; groups were compared using the log‐rank test, and HRs with 95% confidence intervals (CIs) were estimated. The proportional‐hazards assumption was assessed for each outcome and was supported for all outcomes in the primary analysis and the two original sensitivity analyses (proportionality test *p* > 0.05). For the statistically significant primary outcome, we calculated an *E*‐value to quantify the minimum strength of association, on the risk‐ratio scale, that an unmeasured confounder would need with both exposure and outcome to fully explain away the observed association; because the primary outcome was rare (< 15%), the HR was treated as approximating the risk ratio [16]. No adjustment for multiple comparisons was made for secondary outcomes or the additional analyses. Their *p* values are presented without correction. Age‐specific estimates were compared using a two‐sided between‐stratum heterogeneity test based on the difference between independent log HRs, with standard errors derived from their 95% CIs. Because this comparison was derived from aggregate estimates rather than a fitted interaction coefficient, it was interpreted as an approximate test of between‐stratum heterogeneity.

## 4. Results

### 4.1. Study Sample and Baseline Characteristics

**Figure 1:**
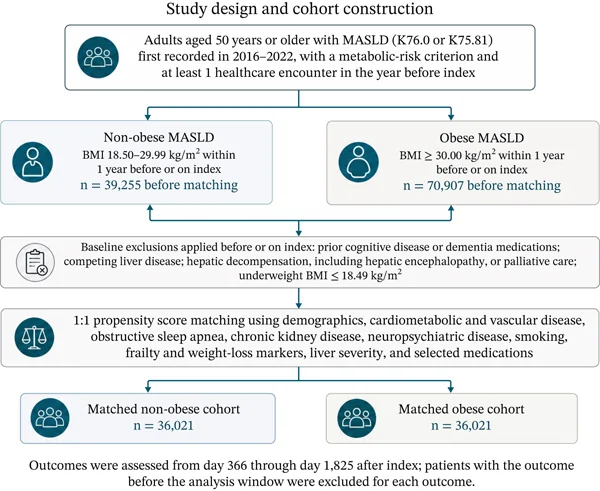
Study design and cohort construction for the primary analysis. Flow diagram of cohort assembly. Eligible patients were adults aged 50 years or older with metabolic dysfunction‐associated steatotic liver disease (MASLD), identified by ICD‐10‐CM codes K76.0 or K75.81 first recorded in 2016–2022, who met at least one metabolic‐risk criterion and had at least one healthcare encounter in the year before the index date. Patients were classified by index body mass index (BMI) as nonobese MASLD (BMI 18.50–29.99 kg/m^2^ within 1 year before or on index; *n* = 39,255 before matching) or obese MASLD (BMI ≥ 30.00 kg/m^2^ within 1 year before or on index; *n* = 70,907 before matching). Baseline exclusions applied before or on index were prior cognitive disease or dementia medications, competing liver disease, hepatic decompensation, including hepatic encephalopathy, or palliative care, and underweight BMI ≤ 18.49 kg/m^2^. Groups underwent 1:1 propensity score matching on demographics, cardiometabolic and vascular disease, obstructive sleep apnea, chronic kidney disease, neuropsychiatric disease, smoking, frailty and weight‐loss markers, liver severity, and selected medications, yielding 36,021 matched patients per cohort. Outcomes were assessed from Day 366 through Day 1825 after index; for each outcome, patients with that outcome before the analysis window were excluded. BMI, body mass index; ICD‐10‐CM, International Classification of Diseases, Tenth Revision, Clinical Modification; MASLD, metabolic dysfunction‐associated steatotic liver disease.

Before matching, we identified 39,255 nonobese and 70,907 obese patients with MASLD meeting eligibility criteria (Figure 1). Before matching, the groups differed on several characteristics: Compared with the nonobese group, obese patients were younger (mean age 62.2 vs. 64.8 years; SMD 0.305) and had a higher prevalence of obstructive sleep apnea (24.4% vs. 9.8%; 0.394), Type 2 diabetes (48.3% vs. 38.7%; 0.195), essential hypertension (78.6% vs. 72.2%; 0.150), and depression (25.1% vs. 20.5%; 0.110), whereas Asian race was more frequent in the nonobese group (6.1% vs. 1.2%; 0.262). After 1:1 propensity score matching, the analytic cohort comprised 36,021 nonobese and 36,021 obese patients (Figure 1 and Table 1), with a mean age of 64.3 years (SD 8.4 and 8.3, respectively) and women comprising 57.0% and 56.9%. Baseline characteristics were well balanced after matching, with all SMDs < 0.10 (Table 1; full matched‐covariate balance in Tables S2, S3, S4).

**Table 1:** Selected baseline characteristics of propensity score–matched nonobese versus obese adults with MASLD.

Characteristic	Nonobese MASLD *N* = 36,021	Obese MASLD *N* = 36,021	SMD
Demographics			
Age at index, mean (SD), year	64.3 (8.4)	64.3 (8.3)	0.001
Female sex	20,547 (57.0)	20,481 (56.9)	0.004
White race	26,169 (72.6)	26,114 (72.5)	0.003
Black or African American race	3544 (9.8)	3570 (9.9)	0.002
Hispanic or Latino ethnicity	6026 (16.7)	6235 (17.3)	0.015
Asian race	864 (2.4)	837 (2.3)	0.005
Cardiometabolic, vascular, sleep, and kidney disease			
Type 2 diabetes mellitus	14,271 (39.6)	14,243 (39.5)	0.002
Essential hypertension	26,325 (73.1)	26,476 (73.5)	0.009
Dyslipidemia	25,061 (69.6)	25,152 (69.8)	0.005
Chronic ischemic heart disease	7971 (22.1)	8113 (22.5)	0.009
Heart failure	2883 (8.0)	2983 (8.3)	0.010
Prior cerebral infarction	1557 (4.3)	1574 (4.4)	0.002
Transient ischemic attack	1207 (3.4)	1189 (3.3)	0.003
Obstructive sleep apnea	3812 (10.6)	3768 (10.5)	0.004
Chronic kidney disease	4057 (11.3)	4237 (11.8)	0.016
Neuropsychiatric, tobacco, frailty, and liver severity			
Depression	7565 (21.0)	7691 (21.4)	0.009
Anxiety disorder	8471 (23.5)	8516 (23.6)	0.003
Personal history of nicotine dependence	7847 (21.8)	7996 (22.2)	0.010
Abnormal weight loss	2063 (5.7)	2046 (5.7)	0.002
Cirrhosis	1125 (3.1)	1134 (3.1)	0.001
Portal hypertension	320 (0.9)	327 (0.9)	0.002
Selected medications			
Atorvastatin	11,021 (30.6)	11,175 (31.0)	0.009
Metformin	9051 (25.1)	9010 (25.0)	0.003
Insulin	7989 (22.2)	8242 (22.9)	0.017
Semaglutide	296 (0.8)	284 (0.8)	0.004
Empagliflozin	911 (2.5)	906 (2.5)	0.001
Sertraline	2644 (7.3)	2755 (7.6)	0.012
Zolpidem	3952 (11.0)	4069 (11.3)	0.010

*Note:* Values are *n* (%) unless otherwise specified. A clinically selected subset of matched covariates is shown; full matched covariate balance is provided in the Supporting Information (Tables S2, S3, S4). All displayed SMDs were < 0.10 after matching.

Abbreviation: SMD = standardized mean difference.

### 4.2. Primary Outcome

**Figure 2:**
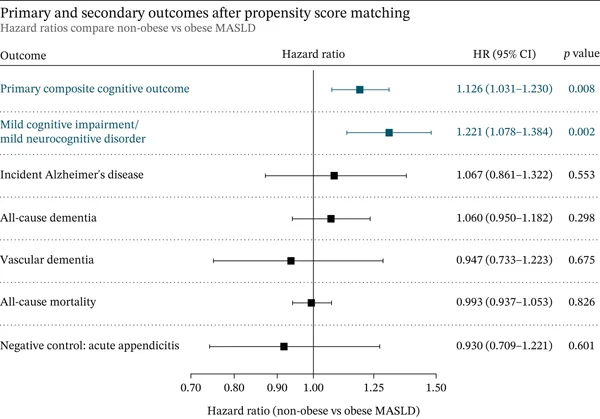
Hazard ratios for incident outcomes in the primary propensity score–matched analysis. Forest plot of hazard ratios from the primary analysis comparing nonobese versus obese MASLD across the primary composite cognitive outcome and the prespecified secondary and control outcomes (mild cognitive impairment/mild neurocognitive disorder, incident Alzheimer disease, all‐cause dementia, vascular dementia, all‐cause mortality, and the negative‐control outcome of acute appendicitis). Squares denote point estimates, and horizontal lines denote 95% confidence intervals; the vertical line indicates a hazard ratio of 1.00. Hazard ratios greater than 1.00 indicate higher risk in nonobese relative to obese MASLD. Outcomes were counted from Day 366 through Day 1825 after index, and patients with the relevant outcome before the analysis window were excluded for that outcome. Corresponding hazard ratios, 95% confidence intervals, and *p* values are reported in Table 2. CI, confidence interval; HR, hazard ratio; MASLD, metabolic dysfunction‐associated steatotic liver disease; MCI, mild cognitive impairment.

Over the prespecified outcome window of Day 366 through Day 1825 after index (1 through 5 years), the primary composite cognitive outcome occurred in 1048 of 35,676 nonobese patients (2.9%) and 939 of 35,768 obese patients (2.6%), an absolute risk difference of +0.3 percentage points. Compared with obese MASLD, nonobese MASLD was associated with a higher risk of the primary composite cognitive outcome (HR 1.126, 95% CI 1.031–1.230; *p* = 0.008) (Figure 2 and Table 2; absolute risks in Figure S2). The *E*‐value for this estimate was 1.50, and the *E*‐value for the confidence limit closest to the null was 1.21, indicating that an unmeasured confounder associated with both nonobese status and the cognitive outcome by a risk ratio of at least 1.50 (1.21 for the lower confidence bound), beyond the matched covariates, would be required to fully explain away the association [16].

**Table 2:** Incident cognitive outcomes, mortality, and negative‐control outcome in propensity score–matched nonobese versus obese adults with MASLD.

Outcome	Nonobese events/at risk (%)	Obese events/at risk (%)	Risk difference (pp)	HR (95% CI)	*p* value
Primary composite cognitive outcome	1048/35,676 (2.9)	939/35,768 (2.6)	+0.3	1.126 (1.031–1.230)	0.008
Mild cognitive impairment/mild neurocognitive disorder	542/35,876 (1.5)	447/35,909 (1.2)	+0.3	1.221 (1.078–1.384)	0.002
Incident Alzheimer disease	172/35,981 (0.5)	162/35,998 (0.5)	0.0	1.067 (0.861–1.322)	0.553
All‐cause dementia	664/35,793 (1.9)	631/35,859 (1.8)	+0.1	1.060 (0.950–1.182)	0.298
Vascular dementia	114/35,982 (0.3)	121/35,989 (0.3)	0.0	0.947 (0.733–1.223)	0.675
All‐cause mortality	2236/34,841 (6.4)	2261/34,880 (6.5)	−0.1	0.993 (0.937–1.053)	0.826
Negative control: acute appendicitis	100/35,707 (0.3)	108/35,722 (0.3)	0.0	0.930 (0.709–1.221)	0.601

*Note:* *p* values are from log‐rank tests. Hazard ratios compare nonobese versus obese MASLD. Outcomes were counted from Day 366 through Day 1825 after index; patients with the relevant outcome before the analysis window were excluded for that outcome. The primary composite cognitive outcome included G30, F01, F02, F03, G31.83, G31.84, and F06.7.

Abbreviation: pp = percentage points.

### 4.3. Secondary Outcomes

Among the prespecified cognitive components and related outcomes, nonobese MASLD was associated with a higher risk of MCI/mild neurocognitive disorder (542/35,876 [1.5%] vs. 447/35,909 [1.2%]; HR 1.221, 95% CI 1.078–1.384; *p* = 0.002). There was no statistically significant difference between groups for incident AD (172/35,981 [0.5%] vs. 162/35,998 [0.5%]; HR 1.067, 95% CI 0.861–1.322; *p* = 0.553), all‐cause dementia (664/35,793 [1.9%] vs. 631/35,859 [1.8%]; HR 1.060, 95% CI 0.950–1.182; *p* = 0.298), or vascular dementia (114/35,982 [0.3%] vs. 121/35,989 [0.3%]; HR 0.947, 95% CI 0.733–1.223; *p* = 0.675). All‐cause mortality was similar between groups (2236/34,841 [6.4%] vs. 2261/34,880 [6.5%]; HR 0.993, 95% CI 0.937–1.053; *p* = 0.826) (Figure 2 and Table 2).

### 4.4. Negative Control Outcome

In the negative control analysis, there was no statistically significant difference in acute appendicitis between nonobese and obese MASLD (100/35,707 [0.3%] vs. 108/35,722 [0.3%]; HR 0.930, 95% CI 0.709–1.221; *p* = 0.601) (Figure 2 and Table 2), consistent with adequate control of measured and, to the extent testable, residual bias.

### 4.5. Sensitivity Analyses

The association between nonobese MASLD and the primary composite cognitive outcome was consistent in direction across both sensitivity analyses (Table S1 and Figure S1). In the longer follow‐up analysis (index 2016–2020; outcomes 1–10 years; 23,240 matched per group), the estimate remained statistically significant (HR 1.089, 95% CI 1.004–1.181; *p* = 0.039). In the simpler adjustment model (matched on age, sex, race/ethnicity, and tobacco use/history; 38,487 matched per group), the estimate was directionally consistent but not statistically significant (HR 1.080, 95% CI 0.994–1.174; *p* = 0.071). For MCI/mild neurocognitive disorder, the association remained statistically significant in both the longer follow‐up analysis (HR 1.166, 95% CI 1.038–1.310; *p* = 0.010) and the simpler adjustment model (HR 1.190, 95% CI 1.056–1.342; *p* = 0.004). Incident AD was not statistically significant in either sensitivity analysis (longer follow‐up HR 1.130, 95% CI 0.945–1.352, *p* = 0.181; simpler adjustment HR 1.160, 95% CI 0.948–1.419, *p* = 0.149).

### 4.6. Repeat and Additional Analyses

The repeat main analysis matched 100,086 patients per group and yielded estimates similar to the primary analysis for the composite outcome (HR 1.132, 95% CI 1.077–1.191; *p* < 0.001) and MCI (HR 1.210, 95% CI 1.125–1.301; *p* < 0.001). After baseline cirrhosis or portal hypertension was excluded and 95,995 patients per group were rematched, the corresponding estimates remained similar for the composite (HR 1.121, 95% CI 1.064–1.180; *p* < 0.001) and MCI (HR 1.198, 95% CI 1.112–1.290; *p* < 0.001). The repeat analysis yielded higher estimates for AD, all‐cause dementia, and vascular dementia than the primary analysis. Full component results are reported in Table S6, and cohort‐construction details are provided in Table S5. The newly matched cohorts were well balanced, with maximum absolute postmatching SMDs ranging from 0.012 to 0.024. Full postmatching covariate balance for the repeat main analysis is provided in Table S9.

The age 50–64 analysis matched 50,004 patients per group, and the age 65 years or older analysis matched 50,388 per group. The composite HRs were 0.985 (95% CI 0.887–1.095, *p* = 0.785) and 1.111 (95% CI 1.050–1.176, *p* < 0.001), respectively, with a between‐stratum heterogeneity *p* value of 0.050. The corresponding MCI HRs were 0.998 (95% CI 0.873–1.141, *p* = 0.976) and 1.225 (95% CI 1.124–1.334, *p* < 0.001), with a heterogeneity *p* value of 0.012. Heterogeneity was not detected for AD, all‐cause dementia, or vascular dementia (all *p* ≥ 0.148). The proportional‐hazards test was significant for the composite outcome in the age 65 years or older stratum (*p* = 0.011), so this HR represents an average relative hazard over the fixed outcome window. Full age‐stratified results are provided in Table S7.

In the same‐cohort follow‐up analysis, one 2016–2020 cohort with 62,500 patients per group was matched once. Extending the outcome window from years 1 through 5 to years 1 through 10 changed the composite HR from 1.117 to 1.108 and the MCI HR from 1.185 to 1.142. Complete results from the same‐cohort follow‐up analysis are provided in Table S8.

## 5. Discussion

Among 72,042 propensity‐score–matched adults aged 50 years or older with MASLD in the TriNetX Research Network, nonobese disease was associated with a modestly higher risk of incident composite cognitive impairment than obese disease (HR 1.126; 95% CI 1.031–1.230; *p* = 0.008), corresponding to a small absolute difference in 5‐year risk (2.9% vs. 2.6%). This difference was concentrated in MCI, the earliest and most frequently coded stage of cognitive decline, whereas incident AD, all‐cause dementia, and vascular dementia showed no significant between‐group difference. Because both groups met identical MASLD eligibility and a shared metabolic‐risk threshold and differed only in body‐mass category, the comparison isolates body habitus rather than the presence of metabolic liver disease itself. To our knowledge, this is the first large multi‐institutional EHR cohort to compare nonobese with obese MASLD directly for incident, code‐defined cognitive diagnoses, extending Korean and long‐term cohort findings that implicated leaner phenotypes [14, 15]. Our nonobese group spanned normal‐weight through overweight patients and therefore reflects a broader and more clinically common population than the lean phenotype emphasized in earlier work. The absolute difference of 0.3 percentage points corresponds to approximately three additional coded composite outcomes per 1000 patients during the years 1 through 5 outcome window. Given the small absolute difference, the observational design, and the absence of a significant dementia association in the primary analysis, these findings do not support a change in cognitive screening or MASLD treatment solely according to obesity status.

Our findings align with a growing literature linking steatotic liver disease to cognitive outcomes while sharpening its body‐habitus dimension. A nationwide Korean cohort found NAFLD associated with higher dementia risk, with a stronger association among nonobese than obese individuals [17], a pattern that parallels our primary result. A separate nationwide study reported MASLD associated with higher incidence of AD and vascular dementia [8], and a systematic review concluded that people with NAFLD performed worse in some, though not all, cognitive domains [7]. Two recent cohorts specifically implicated leaner phenotypes: greater incident AD in lean than in overweight or obese MASLD [14], and higher AD‐specific mortality in nonobese than obese MASLD over 30 years [15]. Our study differs in population (a large multi‐institutional EHR network rather than national registries or mortality records) and in outcome (incident diagnoses spanning MCI through dementia rather than disease‐specific mortality), which likely explains why our clearest association emerged for MCI rather than for incident AD.

Findings in older populations have been discordant. In a Swedish cohort of adults aged 65 years or older, NAFLD was associated with a higher rate of dementia, with an adjusted HR of 1.38, and the risk was greater among patients with heart disease or stroke [18]. The estimates were similar after patients with cirrhosis were excluded [18]. In contrast, an analysis of initially healthy community‐dwelling older adults found Fatty Liver Index‐defined MASLD associated with a lower rate of incident dementia, with an adjusted HR of 0.63 [19]. Differences in cohort health, disease ascertainment, cardiometabolic burden, and late‐life weight trajectories may contribute to these findings. Prodromal weight loss may lower BMI and improve or resolve steatosis before dementia is diagnosed [19].

Several mechanisms could plausibly link nonobese MASLD to greater cognitive vulnerability. Reviews of liver–brain interactions implicate systemic and neuroinflammation, insulin resistance, and disturbance of the gut–liver–brain axis as candidate pathways connecting hepatic steatosis to cognitive dysfunction [20]. Consistent with an inflammatory contribution, a community‐based study found the association between NAFLD and cognitive impairment stronger among participants with higher inflammatory markers [21], and hepatic steatosis has been associated with lower cerebral brain volume independent of cardiometabolic risk factors [6]. Nonobese and lean steatotic liver disease is increasingly recognized as a distinct metabolic phenotype with its own adverse prognosis [11, 12], including higher liver‐related mortality than nonlean disease [13], a vulnerability that may extend to extrahepatic organs including the brain. A noncausal explanation must also be considered: Weight loss during the preclinical phase of neurodegeneration can lower measured BMI and contributes to the so‐called obesity paradox [9, 10], such that some nonobese patients may already be in a preclinical cognitive phase. We matched on frailty, malnutrition, and abnormal‐weight‐loss markers to mitigate this, but because preclinical weight change can precede a recorded cognitive diagnosis, the 1‐year lag before outcome counting may not fully remove its influence. These interpretations should therefore be regarded as plausible rather than established, as none was tested directly.

The concentration of the primary association in MCI, with nonsignificant estimates for the dementia subtypes in the primary analysis, warrants cautious interpretation. MCI is an earlier and more frequently coded stage of decline, whereas the AD and vascular dementia analyses involved fewer events. The MCI association was consistent across the primary, repeat, and sensitivity analyses. In contrast, estimates for the dementia outcomes were less consistent across the additional analyses. AD and all‐cause dementia were statistically significant in the repeat and hepatic‐exclusion analyses, whereas vascular dementia was statistically significant in the repeat main analysis but not after hepatic exclusion. These component findings should be considered hypothesis‐generating rather than evidence of a differential effect on specific dementia subtypes.

The primary analysis already excluded recorded hepatic decompensation and balanced cirrhosis and portal hypertension through matching. After patients with cirrhosis or portal hypertension were excluded and the cohorts were rematched, the composite and MCI estimates remained similar to the repeat main analysis. This finding argues against diagnosed advanced liver disease as the sole explanation, although covert or unrecorded hepatic encephalopathy cannot be excluded. The age‐stratified results suggested stronger associations in adults aged 65 years or older. Between‐stratum heterogeneity was observed for MCI and was near the conventional significance threshold for the composite, with no evidence of heterogeneity for the dementia subtypes. These findings require confirmation.

The same‐cohort follow‐up analysis also informs the numerical attenuation in the original longer follow‐up analysis. When the index period and matched population were held constant, extending follow‐up produced only modest attenuation for the composite and MCI. An association related partly to early ascertainment or prodromal weight change may become diluted as later events accrue, and a single index BMI may become less representative of body habitus over time. The original longer follow‐up analysis also restricted the index period to 2016–2020 and used a smaller, separately matched cohort. Its numerical difference from the primary estimate therefore cannot be attributed to follow‐up duration alone.

The study has several methodological strengths. Holding MASLD eligibility and a shared metabolic‐risk criterion constant while varying only BMI category isolated body habitus as the contrast of interest. Extensive 1:1 propensity‐score matching (across demographics; cardiometabolic, vascular, sleep, and kidney disease; neuropsychiatric disease; smoking; frailty and weight‐loss markers; liver severity; and selected medications) achieved negligible residual imbalance (all SMDs < 0.10). The primary composite was prespecified; a prespecified negative‐control outcome (acute appendicitis) returned a null result, supporting adequate control of measured and, to the extent testable, residual bias; and an E‐value quantified robustness to unmeasured confounding [16]. Finally, restricting outcomes to incident disease (excluding prevalent cognitive diagnoses and dementia medications) together with a fixed 1‐year lag before outcome counting reduced reverse‐causation and protopathic bias.

These strengths are balanced by important limitations. As an observational analysis, the study cannot establish causation, and the *E*‐value indicates that only a moderately strong unmeasured confounder, associated with both nonobese status and the cognitive outcome by a risk ratio of about 1.5 (1.21 for the lower confidence bound), would be needed to explain the association, leaving residual confounding a real possibility. Analyses relied on de‐identified EHR data from TriNetX, whose diagnosis codes were not independently validated and whose predominantly insured, academic, and acute‐care populations may limit generalizability and introduce misclassification and selection bias [22]. Although the prespecified negative control argues against gross unmeasured confounding and general detection bias, it cannot exclude ascertainment differences specific to cognition: for example, more frequent cognitive evaluation in one group or differential coding of MCI, the most discretionary diagnosis and the principal driver of the composite. By design, the nonobese group included overweight individuals, so the contrast reflects a broad, clinically common phenotype rather than truly lean disease, and finer gradients of body composition were not captured. BMI was ascertained from a single measurement, and steatosis was code‐defined without histologic or imaging confirmation of severity. Accordingly, patients identified through K75.81 should be interpreted as part of a code‐defined MASLD cohort rather than as having histologically confirmed steatohepatitis. Despite matching on frailty and weight‐loss markers, residual reverse causation from preclinical weight loss cannot be excluded [9, 10]. Rare outcomes such as incident AD and vascular dementia were underpowered, and secondary outcomes were not adjusted for multiple comparisons. Finally, in the simpler adjustment model the primary association was directionally consistent but no longer statistically significant (HR 1.080; *p* = 0.071), indicating some sensitivity to model specification; however, the MCI association remained significant across all analyses, and the negative control remained null, suggesting that these limitations are unlikely to account fully for the findings. Medication covariates reflected recorded ingredient concepts rather than verified active use, and the aggregate output did not provide dose, duration, adherence, or indication. TriNetX is updated over time, and the repeat and additional analyses included different and substantially larger cohorts than the primary analysis. Comparisons between the primary and repeat analyses may therefore reflect changes in sample size and network composition.

These results point to several priorities for future research. Prospective studies that confirm and grade steatosis with imaging or biomarkers, incorporate longitudinal cognitive testing, and allow sufficient follow‐up to capture progression to dementia would clarify whether the MCI association reflects an early, potentially modifiable process or preclinical neurodegeneration. External validation in geographically defined populations and in healthcare settings underrepresented in TriNetX is needed given the data source, as is finer characterization of body composition (including visceral adiposity and sarcopenia) that BMI cannot capture. Because nearly half of dementia is attributable to modifiable risk factors [23], studies should also assess whether body composition and metabolic management within MASLD influence cognitive trajectories, and whether targeted cognitive screening benefits nonobese patients with metabolic liver disease.

## 6. Conclusion

Among adults aged 50 years or older with MASLD in the TriNetX Research Network, nonobese status was associated with a modestly higher risk of incident composite cognitive impairment than obese status, driven primarily by MCI, with no significant difference for incident AD, all‐cause dementia, vascular dementia, or all‐cause mortality. Body habitus may therefore help identify heterogeneity in cognitive risk within metabolic liver disease. Given the observational design, modest effect size, and reliance on code‐defined outcomes, however, these findings should be confirmed in prospective studies with biomarker‐ or imaging‐confirmed disease before they inform clinical practice, particularly to resolve the AD‐specific question and the issue of causality.

## Author Contributions

Akram Alnounou: conceptualization, methodology, formal analysis, investigation, data curation, writing—original draft, and writing—review and editing. Mohammad Alabbas: investigation, data curation, and writing—review and editing. Riddhi Machchhar: investigation, validation, and writing—review and editing. EzzEIDien A. Ibrahim: investigation, data curation, and writing—review and editing. Mohammad Alali: formal analysis, investigation, and writing—review and editing. Ahmad Alhusen: investigation, validation, and writing—review and editing. Syed‐Mohammed Jafri: investigation, validation, and writing—review and editing. Basile Njei: conceptualization, methodology, supervision, and writing—review and editing.

## Funding

No funding was received for this manuscript.

## Disclosure

All authors read and approved the final manuscript and agree to be accountable for the work.

## Ethics Statement

Not applicable. This study was a secondary analysis of existing, de‐identified, aggregated data obtained through the TriNetX Research Network. The investigators had no access to identifiable private information, could not ascertain individual patient identities, and had no interaction or intervention with patients. Accordingly, the analysis did not involve human subjects as defined under 45 CFR §46.102(e), and institutional review board review was not required. No ethics committee reviewed the study, and no approval number exists.

## Consent

Not applicable. The study used only existing, de‐identified, aggregated data and involved no interaction or intervention with individuals. Individual informed consent was therefore not required. No identifiable individual participant information is reported.

## Conflicts of Interest

Syed‐Mohammed Jafri reports serving as a speaker/consultant for Gilead, AbbVie, Ironwood, Takeda, and Ipsen; these relationships are disclosed because they could be perceived as potential conflicts of interest, but they had no role in the conception, design, conduct, analysis, interpretation, writing, approval, or decision to submit this manuscript, and no funding or other support for this work was received from these entities. The remaining authors declare no conflicts of interest.

## Supporting information


**Supporting Information** Additional supporting information can be found online in the Supporting Information section. Methods S1 provides the analysis specifications for the primary analysis and the two original sensitivity analyses. Supporting Information 2. Supplementary Methods S2 provides the outcome definitions and analysis windows. Supporting Information 3. Supplementary Methods S3 provides the specifications for the repeat main and additional analyses and the medication‐variable interpretation. Supporting Information 4. Table S1 reports cognitive outcome estimates across the primary and original sensitivity analyses. Supporting Information 5–6. Tables S2–S4 provide full postmatching covariate balance for the primary analysis. Supporting Information 7. Table S5 compares query dates, healthcare organization counts, and cohort construction. Supporting Information 8. Table S6 compares the primary, repeat main, and hepatic‐exclusion analyses. Supporting Information 9. Table S7 reports age‐stratified outcomes and heterogeneity tests. Supporting Information 10. Table S8 reports the same‐cohort outcome‐window comparison. Supporting Information 11. Table S9 provides full postmatching balance for the repeat main analysis. Supporting Information 12. Figure S1: Sensitivity analyses for incident cognitive outcomes in nonobese versus obese adults with MASLD. Supporting Information 13. Figure S2: Absolute event risks for cognitive outcomes in propensity score–matched nonobese versus obese adults with MASLD. STROBE Checklist: Completed STROBE checklist for cohort studies.

## Data Availability

The data analyzed in this study were obtained from the TriNetX Research Network and were accessed under a data use agreement. Because the analyses were performed on de‐identified, aggregated patient data within the TriNetX platform, the underlying patient‐level data are not publicly available and cannot be shared by the authors owing to the terms of the TriNetX data use agreement and licensing restrictions. Access to the TriNetX network can be requested directly from TriNetX, LLC (https://trinetx.com/), subject to a data use agreement. The cohort definitions, diagnosis and medication code lists, outcome definitions, and analysis specifications required to reproduce the analyses are provided in the Supporting Information.
