# Supplementary material for: Incident Cognitive Impairment in Nonobese Versus Obese Adults Aged 50 Years or Older With Metabolic Dysfunction‐Associated Steatotic Liver Disease: A Propensity Score–Matched Cohort Study
Source: Int J Hepatol. 2026 Aug 26;2026:7550224. doi: 10.1155/ijh/7550224 (PMC13509417; doi:10.1155/ijh/7550224)
Supplement: Supplementary file 1 [file IJH-2026-7550224-s001.zip › Alnounou_MASLD_Supplement_Final 2.docx]

**SUPPLEMENTARY MATERIAL**

**Incident Cognitive Impairment in Non-Obese Versus Obese Adults Aged 50 Years or Older With Metabolic Dysfunction-Associated Steatotic Liver Disease: A Propensity Score–Matched Cohort Study**

Akram Alnounou et al.

**Supplementary Methods S1. Analysis specifications for primary and sensitivity analyses**

| **Analysis** | **Why it was run** | **Index period** | **Outcome window** | **Matching / adjustment** |
| --- | --- | --- | --- | --- |
| Main analysis | Primary matched comparison of non-obese versus obese MASLD. | 2016-2022 | Day 366-1,825 | Full covariate matching across demographics, cardiometabolic and vascular disease, obstructive sleep apnea, chronic kidney disease, neuropsychiatric disease, smoking, frailty and weight-loss markers, liver severity, and selected medications. |
| Longer follow-up analysis | Tests whether estimates change when earlier-indexed patients have a longer available outcome window. | 2016-2020 | Day 366-3,650 | Same full clinical matching approach as the main analysis. |
| Simpler adjustment model | Tests whether extensive clinical matching materially attenuates estimates. | 2016-2022 | Day 366-1,825 | Matched only on age, sex, race/ethnicity, and tobacco use/history. |

*All analyses used the same exposure definition: age >=50 years at first MASLD code, non-obese BMI 18.50-29.99 kg/m2 versus obese BMI >=30.00 kg/m2 within 1 year before/on index, underweight BMI <=18.49 kg/m^2^ excluded, baseline metabolic-risk criterion, prior cognitive/liver/decompensation exclusions, and baseline healthcare-contact requirement.*

**Supplementary Methods S2. Outcome definitions and analysis windows**

| **Outcome** | **Code-defined terms** | **Analysis window** |
| --- | --- | --- |
| Alzheimer disease | G30, G30.0, G30.1, G30.8, G30.9 | Day 366-1,825 after index |
| All-cause dementia | G30, F01, F02, F03, G31.83 | Day 366-1,825 after index |
| Vascular dementia | F01 | Day 366-1,825 after index |
| Mild cognitive impairment / mild neurocognitive disorder | G31.84, F06.7 | Day 366-1,825 after index |
| Primary composite cognitive outcome | G30, F01, F02, F03, G31.83, G31.84, F06.7 | Day 366-1,825 after index |
| All-cause mortality | TriNetX Deceased term | Day 366-1,825 after index |
| Negative control: acute appendicitis | K35 | Day 366-1,825 after index |

*Patients with the relevant outcome before the analysis window were excluded separately for each outcome analysis.*

**Table S1. Cognitive outcome estimates across primary and sensitivity analyses**

| **Outcome** | **Analysis** | **Index period / outcome window** | **Matched N per cohort** | **HR (95% CI)** | **P value** |
| --- | --- | --- | --- | --- | --- |
| **Primary composite cognitive outcome** | | | | | |
|  | Main analysis | 2016-2022; 1-5 years | 36,021 | 1.126 (1.031-1.230) | 0.008 |
|  | Longer follow-up analysis | 2016-2020; 1-10 years | 23,240 | 1.089 (1.004-1.181) | 0.039 |
|  | Simpler adjustment model | 2016-2022; 1-5 years | 38,487 | 1.080 (0.994-1.174) | 0.071 |
| **Mild cognitive impairment / mild neurocognitive disorder** | | | | | |
|  | Main analysis | 2016-2022; 1-5 years | 36,021 | 1.221 (1.078-1.384) | 0.002 |
|  | Longer follow-up analysis | 2016-2020; 1-10 years | 23,240 | 1.166 (1.038-1.310) | 0.010 |
|  | Simpler adjustment model | 2016-2022; 1-5 years | 38,487 | 1.190 (1.056-1.342) | 0.004 |
| **Incident Alzheimer disease** | | | | | |
|  | Main analysis | 2016-2022; 1-5 years | 36,021 | 1.067 (0.861-1.322) | 0.553 |
|  | Longer follow-up analysis | 2016-2020; 1-10 years | 23,240 | 1.130 (0.945-1.352) | 0.181 |
|  | Simpler adjustment model | 2016-2022; 1-5 years | 38,487 | 1.160 (0.948-1.419) | 0.149 |

*The simpler adjustment model is included only as a sensitivity analysis; it matched on age, sex, race/ethnicity, and tobacco use/history.*

**Table S2. Full postmatching covariate balance for demographic, cardiometabolic, vascular, sleep, and kidney-disease variables**

| **Domain** | **Characteristic** | **Non-obese MASLD** | **Obese MASLD** | **SMD** |
| --- | --- | --- | --- | --- |
| Demographics | Age at index, mean (SD), y | 64.3 +/- 8.4 | 64.3 +/- 8.3 | 0.001 |
| Demographics | White race | 26,169 (72.6) | 26,114 (72.5) | 0.003 |
| Demographics | Female sex | 20,547 (57.0) | 20,481 (56.9) | 0.004 |
| Demographics | Not Hispanic or Latino ethnicity | 24,346 (67.6) | 24,274 (67.4) | 0.004 |
| Demographics | Hispanic or Latino ethnicity | 6,026 (16.7) | 6,235 (17.3) | 0.015 |
| Demographics | Black or African American race | 3,544 (9.8) | 3,570 (9.9) | 0.002 |
| Demographics | Male sex | 15,472 (43.0) | 15,533 (43.1) | 0.003 |
| Demographics | Other race | 2,494 (6.9) | 2,532 (7.0) | 0.004 |
| Demographics | Asian race | 864 (2.4) | 837 (2.3) | 0.005 |
| Diagnosis | Type 2 diabetes mellitus | 14,271 (39.6) | 14,243 (39.5) | 0.002 |
| Diagnosis | Essential hypertension | 26,325 (73.1) | 26,476 (73.5) | 0.009 |
| Diagnosis | Hypertensive heart disease | 1,946 (5.4) | 1,992 (5.5) | 0.006 |
| Diagnosis | Hypertensive chronic kidney disease | 2,332 (6.5) | 2,493 (6.9) | 0.018 |
| Diagnosis | Hypertensive heart and chronic kidney disease | 650 (1.8) | 735 (2.0) | 0.017 |
| Diagnosis | Secondary hypertension | 640 (1.8) | 631 (1.8) | 0.002 |
| Diagnosis | Hypertensive crisis | 544 (1.5) | 568 (1.6) | 0.005 |
| Diagnosis | Dyslipidemia | 25,061 (69.6) | 25,152 (69.8) | 0.005 |
| Diagnosis | Chronic ischemic heart disease | 7,971 (22.1) | 8,113 (22.5) | 0.009 |
| Diagnosis | Acute myocardial infarction | 1,658 (4.6) | 1,713 (4.8) | 0.007 |
| Diagnosis | Subsequent myocardial infarction | 19 (0.1) | 23 (0.1) | 0.005 |
| Diagnosis | Old myocardial infarction | 1,999 (5.5) | 2,042 (5.7) | 0.005 |
| Diagnosis | Heart failure | 2,883 (8.0) | 2,983 (8.3) | 0.010 |
| Diagnosis | Atrial fibrillation and flutter | 2,814 (7.8) | 2,883 (8.0) | 0.007 |
| Diagnosis | Prior cerebral infarction | 1,557 (4.3) | 1,574 (4.4) | 0.002 |
| Diagnosis | Peripheral vascular disease | 2,288 (6.4) | 2,325 (6.5) | 0.004 |
| Diagnosis | Transient ischemic attack | 1,207 (3.4) | 1,189 (3.3) | 0.003 |
| Diagnosis | Obstructive sleep apnea | 3,812 (10.6) | 3,768 (10.5) | 0.004 |
| Diagnosis | Chronic kidney disease | 4,057 (11.3) | 4,237 (11.8) | 0.016 |

*SMD = standardized mean difference. This table displays the complete set of non-medication covariates from the demographics through kidney-disease domains in the primary full clinical matching model.*

**Table S3. Full postmatching covariate balance for neuropsychiatric, tobacco, frailty, weight-loss, and liver-severity variables**

| **Domain** | **Characteristic** | **Non-obese MASLD** | **Obese MASLD** | **SMD** |
| --- | --- | --- | --- | --- |
| Diagnosis | Depression | 7,565 (21.0) | 7,691 (21.4) | 0.009 |
| Diagnosis | Recurrent major depression | 1,717 (4.8) | 1,798 (5.0) | 0.010 |
| Diagnosis | Anxiety disorder | 8,471 (23.5) | 8,516 (23.6) | 0.003 |
| Diagnosis | Psychotic disorder | 461 (1.3) | 482 (1.3) | 0.005 |
| Diagnosis | Bipolar disorder | 692 (1.9) | 714 (2.0) | 0.004 |
| Diagnosis | Intracranial injury | 694 (1.9) | 691 (1.9) | 0.001 |
| Diagnosis | Epilepsy and recurrent seizures | 702 (1.9) | 686 (1.9) | 0.003 |
| Diagnosis | Convulsions, not elsewhere classified | 801 (2.2) | 792 (2.2) | 0.002 |
| Diagnosis | Parkinson's disease | 224 (0.6) | 225 (0.6) | <0.001 |
| Diagnosis | Secondary parkinsonism | 37 (0.1) | 44 (0.1) | 0.006 |
| Diagnosis | Nicotine dependence | 5,465 (15.2) | 5,568 (15.5) | 0.008 |
| Diagnosis | Tobacco use | 2,951 (8.2) | 2,996 (8.3) | 0.005 |
| Diagnosis | Personal history of nicotine dependence | 7,847 (21.8) | 7,996 (22.2) | 0.010 |
| Diagnosis | Physical debility | 50 (0.1) | 55 (0.2) | 0.004 |
| Diagnosis | Sarcopenia | 10 (0.0) | 10 (0.0) | <0.001 |
| Diagnosis | Severe protein-calorie malnutrition | 193 (0.5) | 204 (0.6) | 0.004 |
| Diagnosis | Moderate/mild protein-calorie malnutrition | 250 (0.7) | 255 (0.7) | 0.002 |
| Diagnosis | Unspecified protein-calorie malnutrition | 344 (1.0) | 318 (0.9) | 0.008 |
| Diagnosis | Abnormal weight loss | 2,063 (5.7) | 2,046 (5.7) | 0.002 |
| Diagnosis | Cachexia | 30 (0.1) | 18 (0.0) | 0.013 |
| Diagnosis | Hepatic fibrosis | 401 (1.1) | 401 (1.1) | <0.001 |
| Diagnosis | Cirrhosis | 1,125 (3.1) | 1,134 (3.1) | 0.001 |
| Diagnosis | Portal hypertension | 320 (0.9) | 327 (0.9) | 0.002 |

*SMD = standardized mean difference. All listed variables were included in the primary full clinical matching model.*

**Table S4. Full postmatching covariate balance for selected recorded medication characteristics**

| **Medication** | **Non-obese MASLD** | **Obese MASLD** | **SMD** |
| --- | --- | --- | --- |
| Atorvastatin | 11,021 (30.6) | 11,175 (31.0) | 0.009 |
| Metformin | 9,051 (25.1) | 9,010 (25.0) | 0.003 |
| Rosuvastatin | 4,481 (12.4) | 4,529 (12.6) | 0.004 |
| Simvastatin | 5,366 (14.9) | 5,383 (14.9) | 0.001 |
| Pravastatin | 3,292 (9.1) | 3,315 (9.2) | 0.002 |
| Insulin | 7,989 (22.2) | 8,242 (22.9) | 0.017 |
| Semaglutide | 296 (0.8) | 284 (0.8) | 0.004 |
| Liraglutide | 552 (1.5) | 580 (1.6) | 0.006 |
| Dulaglutide | 659 (1.8) | 717 (2.0) | 0.012 |
| Tirzepatide | 10 (0.0) | 10 (0.0) | <0.001 |
| Empagliflozin | 911 (2.5) | 906 (2.5) | 0.001 |
| Dapagliflozin | 407 (1.1) | 417 (1.2) | 0.003 |
| Canagliflozin | 448 (1.2) | 465 (1.3) | 0.004 |
| Sertraline | 2,644 (7.3) | 2,755 (7.6) | 0.012 |
| Escitalopram | 2,681 (7.4) | 2,721 (7.6) | 0.004 |
| Fluoxetine | 1,522 (4.2) | 1,512 (4.2) | 0.001 |
| Venlafaxine | 1,571 (4.4) | 1,588 (4.4) | 0.002 |
| Duloxetine | 2,126 (5.9) | 2,200 (6.1) | 0.009 |
| Bupropion | 1,945 (5.4) | 1,969 (5.5) | 0.003 |
| Mirtazapine | 951 (2.6) | 964 (2.7) | 0.002 |
| Trazodone | 3,214 (8.9) | 3,334 (9.3) | 0.012 |
| Alprazolam | 3,326 (9.2) | 3,394 (9.4) | 0.006 |
| Lorazepam | 5,398 (15.0) | 5,528 (15.3) | 0.010 |
| Clonazepam | 2,014 (5.6) | 2,027 (5.6) | 0.002 |
| Diazepam | 3,070 (8.5) | 3,140 (8.7) | 0.007 |
| Temazepam | 784 (2.2) | 775 (2.2) | 0.002 |
| Zolpidem | 3,952 (11.0) | 4,069 (11.3) | 0.010 |
| Eszopiclone | 279 (0.8) | 282 (0.8) | 0.001 |

SMD = standardized mean difference. All listed medication terms were included in the primary full clinical matching model. Medication variables indicate the presence of a recorded RxNorm ingredient concept used as an EHR matching characteristic. The aggregate output does not establish active or concurrent use, dispensing, dose, duration, adherence, or indication. A patient could contribute to more than one medication row.

**Supplementary Methods S3. Repeat main analysis, additional analyses, and medication-variable interpretation**
Because TriNetX is updated over time, the main analysis was repeated using the same query date and network as the additional analyses. The repeat analysis used the same 2016–2022 index period, day 366 through day 1,825 outcome window, eligibility criteria, outcome definitions, and full clinical matching approach as the primary analysis. Exact query dates and network counts are provided in Table S5. The hepatic-exclusion sensitivity analysis additionally excluded cirrhosis (K74.6) or portal hypertension (K76.6) before or on index. Cohorts were rebuilt and rematched, and cirrhosis and portal hypertension were omitted from that propensity model because both were absent by design. The same-cohort follow-up analysis used one 2016–2020 matched cohort for outcome windows from day 366 through day 1,825 and day 366 through day 3,650.
Age strata were nonoverlapping at 50 to 64 years and 65 years or older. Each stratum was independently rematched using the full clinical model. Standard errors were calculated as the natural logarithm of the upper confidence limit minus the natural logarithm of the lower confidence limit, divided by 3.92. The z statistic was the difference between the two log HRs divided by the square root of the sum of their squared standard errors. Two-sided P values were calculated from the standard normal distribution. These tests approximate between-stratum heterogeneity because they were derived from aggregate HRs and CIs rather than coefficients from a fitted exposure-by-age interaction model. No adjustment was made for multiple comparisons.
Medication matching variables indicate the presence of recorded RxNorm ingredient concepts in the aggregate TriNetX output. They do not establish active or concurrent use, dispensing, dose, duration, adherence, or indication. A patient could contribute to more than one medication row. The frequencies in Table S4 should therefore not be interpreted as point-prevalence estimates of active prescribing.

**Table S5. Query date and cohort-construction comparison**

| **Analysis** | Query date | **HCOs available** | **Index period / outcome window** | **Query-defined N, non-obese / obese** | **Entered PSM, non-obese / obese** | **Matched N, non-obese / obese** |
| --- | --- | --- | --- | --- | --- | --- |
| Primary analysis | June 12, 2026 | 111 | 2016–2022 / days 366–1,825 | Not separately available | 39,255 / 70,907 | 36,021 / 36,021 |
| Repeat main analysis | July 29, 2026 | 115 | 2016–2022 / days 366–1,825 | 114,044 / 221,108 | 109,789 / 211,873 | 100,086 / 100,086 |
| Hepatic-exclusion sensitivity | July 29, 2026 | 115 | 2016–2022 / days 366–1,825 | 109,508 / 210,745 | 105,496 / 202,160 | 95,995 / 95,995 |
| Age 50–64 years | July 29, 2026 | 115 online* | 2016–2022 / days 366–1,825 | Not reported in export | 52,641 / 129,172 | 50,004 / 50,004 |
| Age ≥65 years | July 29, 2026 | 115 | 2016–2022 / days 366–1,825 | 59,887 / 87,272 | 57,505 / 83,561 | 50,388 / 50,388 |
| Same-cohort follow-up | July 29, 2026 | 115 | 2016–2020 / days 366–1,825 and 366–3,650 | 70,882 / 140,818 | 68,071 / 134,664 | 62,500 / 62,500 |

PSM = propensity-score matching. The primary analysis output reported the numbers entering matching but did not report a separate query-defined total. The age 50 to 64 export reported 118 healthcare organizations in the network and 115 online for analysis. The repeat main analysis and all additional analyses used the same query date and network. The original analysis remained the primary analysis reported in the abstract, main tables, and figures. Maximum absolute postmatching SMDs were 0.018 for the primary analysis, 0.016 for the repeat main analysis, 0.017 for the hepatic-exclusion sensitivity analysis, 0.022 for the age 50 to 64 analysis, 0.012 for the age 65 years or older analysis, and 0.024 for the same-cohort follow-up analysis.

**Table S6. Outcome estimates across the primary, repeat main, and hepatic-exclusion analyses**

| **Outcome** | **Analysis** | **Matched N per group** | **Non-obese events/at risk** | **Obese events/at risk** | **HR (95% CI)** | **Log-rank P** | **PH P** | **Risk difference, pp** |
| --- | --- | --- | --- | --- | --- | --- | --- | --- |
| **Composite** |  |  |  |  |  |  |  |  |
|  | Primary analysis | 36,021 | 1,048/35,676 | 939/35,768 | 1.126 (1.031-1.230) | 0.008 | >0.05 | +0.31 |
|  | Repeat main analysis | 100,086 | 3,238/99,007 | 2,902/99,224 | 1.132 (1.077-1.191) | <0.001 | 0.101 | +0.35 |
|  | Hepatic-exclusion sensitivity | 95,995 | 3,059/94,990 | 2,769/95,165 | 1.121 (1.064-1.180) | <0.001 | 0.262 | +0.31 |
| **MCI / mild neurocognitive disorder** |  |  |  |  |  |  |  |  |
|  | Primary analysis | 36,021 | 542/35,876 | 447/35,909 | 1.221 (1.078-1.384) | 0.002 | >0.05 | +0.27 |
|  | Repeat main analysis | 100,086 | 1,594/99,638 | 1,335/99,732 | 1.210 (1.125-1.301) | <0.001 | 0.347 | +0.26 |
|  | Hepatic-exclusion sensitivity | 95,995 | 1,525/95,578 | 1,290/95,660 | 1.198 (1.112-1.290) | <0.001 | 0.668 | +0.25 |
| **Alzheimer disease** |  |  |  |  |  |  |  |  |
|  | Primary analysis | 36,021 | 172/35,981 | 162/35,998 | 1.067 (0.861-1.322) | 0.553 | >0.05 | +0.03 |
|  | Repeat main analysis | 100,086 | 660/99,972 | 513/99,991 | 1.302 (1.161-1.462) | <0.001 | 0.348 | +0.15 |
|  | Hepatic-exclusion sensitivity | 95,995 | 638/95,889 | 509/95,910 | 1.269 (1.129-1.426) | <0.001 | 0.079 | +0.13 |
| **All-cause dementia** |  |  |  |  |  |  |  |  |
|  | Primary analysis | 36,021 | 664/35,793 | 631/35,859 | 1.060 (0.950-1.182) | 0.298 | >0.05 | +0.10 |
|  | Repeat main analysis | 100,086 | 2,237/99,369 | 2,019/99,515 | 1.123 (1.057-1.192) | <0.001 | 0.092 | +0.22 |
|  | Hepatic-exclusion sensitivity | 95,995 | 2,110/95,330 | 1,930/95,446 | 1.108 (1.041-1.178) | 0.001 | 0.108 | +0.19 |
| **Vascular dementia** |  |  |  |  |  |  |  |  |
|  | Primary analysis | 36,021 | 114/35,982 | 121/35,989 | 0.947 (0.733-1.223) | 0.675 | >0.05 | -0.02 |
|  | Repeat main analysis | 100,086 | 434/99,961 | 380/99,977 | 1.155 (1.006-1.325) | 0.040 | 0.533 | +0.05 |
|  | Hepatic-exclusion sensitivity | 95,995 | 422/95,874 | 387/95,892 | 1.103 (0.961-1.266) | 0.165 | 0.217 | +0.04 |
| **All-cause mortality** |  |  |  |  |  |  |  |  |
|  | Primary analysis | 36,021 | 2,236/34,841 | 2,261/34,880 | 0.993 (0.937-1.053) | 0.826 | >0.05 | -0.06 |
|  | Repeat main analysis | 100,086 | 6,703/96,887 | 6,674/97,059 | 1.014 (0.980-1.049) | 0.424 | 0.001 | +0.04 |
|  | Hepatic-exclusion sensitivity | 95,995 | 6,040/93,107 | 6,048/93,320 | 1.009 (0.973-1.045) | 0.636 | <0.001 | +0.01 |
| **Acute appendicitis (negative control)** |  |  |  |  |  |  |  |  |
|  | Primary analysis | 36,021 | 100/35,707 | 108/35,722 | 0.930 (0.709-1.221) | 0.601 | >0.05 | -0.02 |
|  | Repeat main analysis | 100,086 | 275/99,140 | 287/99,160 | 0.968 (0.820-1.142) | 0.697 | 0.115 | -0.01 |
|  | Hepatic-exclusion sensitivity | 95,995 | 258/95,060 | 287/95,096 | 0.908 (0.768-1.074) | 0.261 | 0.271 | -0.03 |

Hazard ratios compare non-obese versus obese MASLD. PH P is the proportional-hazards test P value. Primary-analysis PH tests were all greater than 0.05. Hepatic exclusion removed baseline cirrhosis or portal hypertension before the cohorts were rebuilt and rematched.

**Table S7. Age-stratified outcome estimates and between-stratum heterogeneity**

| **Outcome** | **Age stratum** | **Matched N per group** | **Non-obese events/at risk** | **Obese events/at risk** | **HR (95% CI)** | **Log-rank P** | **PH P** | **P heterogeneity** |
| --- | --- | --- | --- | --- | --- | --- | --- | --- |
| **Composite** |  |  |  |  |  |  |  |  |
|  | Age 50–64 years | 50,004 | 681/49,778 | 698/49,820 | 0.985 (0.887-1.095) | 0.785 | 0.797 | 0.050 |
|  | Age ≥65 years | 50,388 | 2,541/49,525 | 2,322/49,700 | 1.111 (1.050-1.176) | <0.001 | 0.011 | 0.050 |
| **MCI / mild neurocognitive disorder** |  |  |  |  |  |  |  |  |
|  | Age 50–64 years | 50,004 | 425/49,885 | 430/49,905 | 0.998 (0.873-1.141) | 0.976 | 0.942 | 0.012 |
|  | Age ≥65 years | 50,388 | 1,164/50,058 | 963/50,125 | 1.225 (1.124-1.334) | <0.001 | 0.166 | 0.012 |
| **Alzheimer disease** |  |  |  |  |  |  |  |  |
|  | Age 50–64 years | 50,004 | 78/49,988 | 65/49,989 | 1.212 (0.872-1.684) | 0.252 | 0.356 | 0.873 |
|  | Age ≥65 years | 50,388 | 571/50,288 | 463/50,312 | 1.247 (1.103-1.410) | <0.001 | 0.410 | 0.873 |
| **All-cause dementia** |  |  |  |  |  |  |  |  |
|  | Age 50–64 years | 50,004 | 344/49,881 | 359/49,908 | 0.967 (0.834-1.122) | 0.661 | 0.621 | 0.148 |
|  | Age ≥65 years | 50,388 | 1,877/49,786 | 1,745/49,919 | 1.090 (1.021-1.163) | 0.010 | 0.053 | 0.148 |
| **Vascular dementia** |  |  |  |  |  |  |  |  |
|  | Age 50–64 years | 50,004 | 61/49,981 | 69/49,985 | 0.892 (0.632-1.259) | 0.515 | 0.419 | 0.279 |
|  | Age ≥65 years | 50,388 | 367/50,286 | 338/50,305 | 1.097 (0.946-1.271) | 0.221 | 0.751 | 0.279 |
| **All-cause mortality** |  |  |  |  |  |  |  |  |
|  | Age 50–64 years | 50,004 | 2,162/48,898 | 2,070/49,007 | 1.054 (0.992-1.119) | 0.088 | 0.122 | 0.104 |
|  | Age ≥65 years | 50,388 | 4,542/48,285 | 4,615/48,356 | 0.992 (0.952-1.033) | 0.686 | 0.012 | 0.104 |
| **Acute appendicitis (negative control)** |  |  |  |  |  |  |  |  |
|  | Age 50–64 years | 50,004 | 158/49,442 | 178/49,459 | 0.895 (0.722-1.109) | 0.309 | 0.567 | 0.941 |
|  | Age ≥65 years | 50,388 | 115/49,988 | 128/50,006 | 0.906 (0.705-1.166) | 0.444 | 0.068 | 0.941 |

The two age strata were independently rematched and are disjoint. P heterogeneity compares the two log HRs using standard errors derived from their 95% CIs. These P values are approximate because they were calculated from aggregate HRs and CIs rather than a fitted interaction model. The proportionality test for the composite in the age 65 years or older stratum was P = 0.011, so that HR represents an average relative hazard over the fixed outcome window.

**Table S8. Same-cohort follow-up analysis comparing years 1 through 5 and years 1 through 10 outcome windows**

| **Outcome** | **Outcome window** | **Matched N per group** | **Non-obese events/at risk** | **Obese events/at risk** | **HR (95% CI)** | **Log-rank P** | **PH P** |
| --- | --- | --- | --- | --- | --- | --- | --- |
| **Composite** |  |  |  |  |  |  |  |
|  | Years 1–5 (days 366–1,825) | 62,500 | 2,110/61,877 | 1,909/61,962 | 1.117 (1.050-1.188) | <0.001 | 0.033 |
|  | Years 1–10 (days 366–3,650) | 62,500 | 3,744/61,877 | 3,429/61,962 | 1.108 (1.058-1.160) | <0.001 | 0.330 |
| **MCI / mild neurocognitive disorder** |  |  |  |  |  |  |  |
|  | Years 1–5 (days 366–1,825) | 62,500 | 1,002/62,242 | 853/62,271 | 1.185 (1.082-1.299) | <0.001 | 0.229 |
|  | Years 1–10 (days 366–3,650) | 62,500 | 1,815/62,242 | 1,611/62,271 | 1.142 (1.068-1.221) | <0.001 | 0.124 |
| **Alzheimer disease** |  |  |  |  |  |  |  |
|  | Years 1–5 (days 366–1,825) | 62,500 | 424/62,435 | 351/62,448 | 1.217 (1.057-1.402) | 0.006 | 0.351 |
|  | Years 1–10 (days 366–3,650) | 62,500 | 860/62,435 | 686/62,448 | 1.269 (1.148-1.403) | <0.001 | 0.717 |
| **All-cause dementia** |  |  |  |  |  |  |  |
|  | Years 1–5 (days 366–1,825) | 62,500 | 1,463/62,080 | 1,354/62,150 | 1.090 (1.013-1.174) | 0.022 | 0.068 |
|  | Years 1–10 (days 366–3,650) | 62,500 | 2,708/62,080 | 2,483/62,150 | 1.105 (1.046-1.166) | <0.001 | 0.933 |
| **Vascular dementia** |  |  |  |  |  |  |  |
|  | Years 1–5 (days 366–1,825) | 62,500 | 302/62,425 | 292/62,427 | 1.042 (0.887-1.224) | 0.619 | 0.357 |
|  | Years 1–10 (days 366–3,650) | 62,500 | 560/62,425 | 532/62,427 | 1.064 (0.945-1.198) | 0.307 | 0.762 |
| **All-cause mortality** |  |  |  |  |  |  |  |
|  | Years 1–5 (days 366–1,825) | 62,500 | 4,701/60,568 | 4,728/60,690 | 1.000 (0.961-1.042) | 0.981 | 0.005 |
|  | Years 1–10 (days 366–3,650) | 62,500 | 7,439/60,568 | 7,644/60,690 | 0.981 (0.950-1.013) | 0.243 | 0.049 |
| **Acute appendicitis (negative control)** |  |  |  |  |  |  |  |
|  | Years 1–5 (days 366–1,825) | 62,500 | 186/61,944 | 199/61,929 | 0.941 (0.770-1.149) | 0.548 | 0.873 |
|  | Years 1–10 (days 366–3,650) | 62,500 | 289/61,944 | 309/61,929 | 0.944 (0.804-1.108) | 0.479 | 0.718 |

Both rows for each outcome use the same 2016–2020 cohort, matched once with 62,500 patients per group. This holds the index period and matched population constant while changing the outcome window. PH P is the proportional-hazards test P value. For rows with PH P < 0.05, HRs represent average relative hazards over the specified outcome window.

**Table S9. Full postmatching covariate balance for the repeat main analysis**

| **Domain** | **Characteristic** | **Non-obese MASLD N=100,086** | **Obese MASLD N=100,086** | **SMD** |
| --- | --- | --- | --- | --- |
| Demographics | Age at index, mean (SD), y | 64.6 ± 8.4 | 64.6 ± 8.3 | <0.001 |
| Demographics | White race | 78,572 (78.5) | 78,702 (78.6) | 0.003 |
| Demographics | Female sex | 57,119 (57.1) | 56,726 (56.7) | 0.008 |
| Demographics | Not Hispanic or Latino ethnicity | 77,235 (77.2) | 77,146 (77.1) | 0.002 |
| Demographics | Hispanic or Latino ethnicity | 9,385 (9.4) | 9,719 (9.7) | 0.011 |
| Demographics | Black or African American race | 9,198 (9.2) | 9,100 (9.1) | 0.003 |
| Demographics | Male sex | 42,966 (42.9) | 43,353 (43.3) | 0.008 |
| Demographics | Other race | 2,799 (2.8) | 2,830 (2.8) | 0.002 |
| Demographics | Asian race | 3,888 (3.9) | 3,730 (3.7) | 0.008 |
| Diagnosis | Type 2 diabetes mellitus | 38,065 (38.0) | 38,232 (38.2) | 0.003 |
| Diagnosis | Essential hypertension | 73,390 (73.3) | 73,802 (73.7) | 0.009 |
| Diagnosis | Hypertensive heart disease | 5,467 (5.5) | 5,531 (5.5) | 0.003 |
| Diagnosis | Hypertensive chronic kidney disease | 6,107 (6.1) | 6,434 (6.4) | 0.013 |
| Diagnosis | Hypertensive heart and chronic kidney disease | 1,790 (1.8) | 1,931 (1.9) | 0.010 |
| Diagnosis | Secondary hypertension | 2,420 (2.4) | 2,495 (2.5) | 0.005 |
| Diagnosis | Hypertensive crisis | 1,511 (1.5) | 1,570 (1.6) | 0.005 |
| Diagnosis | Dyslipidemia | 73,073 (73.0) | 73,646 (73.6) | 0.013 |
| Diagnosis | Chronic ischemic heart disease | 21,586 (21.6) | 22,036 (22.0) | 0.011 |
| Diagnosis | Acute myocardial infarction | 4,736 (4.7) | 4,869 (4.9) | 0.006 |
| Diagnosis | Subsequent myocardial infarction | 60 (0.1) | 68 (0.1) | 0.003 |
| Diagnosis | Old myocardial infarction | 5,488 (5.5) | 5,671 (5.7) | 0.008 |
| Diagnosis | Heart failure | 7,879 (7.9) | 8,143 (8.1) | 0.010 |
| Diagnosis | Atrial fibrillation and flutter | 8,375 (8.4) | 8,622 (8.6) | 0.009 |
| Diagnosis | Prior cerebral infarction | 4,457 (4.5) | 4,646 (4.6) | 0.009 |
| Diagnosis | Peripheral vascular disease | 6,820 (6.8) | 6,942 (6.9) | 0.005 |
| Diagnosis | Transient ischemic attack | 3,680 (3.7) | 3,768 (3.8) | 0.005 |
| Diagnosis | Obstructive sleep apnea | 12,427 (12.4) | 12,384 (12.4) | 0.001 |
| Diagnosis | Chronic kidney disease | 11,292 (11.3) | 11,615 (11.6) | 0.010 |
| Diagnosis | Depression | 21,505 (21.5) | 21,750 (21.7) | 0.006 |
| Diagnosis | Recurrent major depression | 5,125 (5.1) | 5,233 (5.2) | 0.005 |
| Diagnosis | Anxiety disorder | 25,307 (25.3) | 25,358 (25.3) | 0.001 |
| Diagnosis | Psychotic disorder | 1,245 (1.2) | 1,283 (1.3) | 0.003 |
| Diagnosis | Bipolar disorder | 1,972 (2.0) | 2,031 (2.0) | 0.004 |
| Diagnosis | Intracranial injury | 2,090 (2.1) | 2,151 (2.1) | 0.004 |
| Diagnosis | Epilepsy and recurrent seizures | 1,773 (1.8) | 1,816 (1.8) | 0.003 |
| Diagnosis | Convulsions, not elsewhere classified | 2,186 (2.2) | 2,277 (2.3) | 0.006 |
| Diagnosis | Parkinson's disease | 530 (0.5) | 546 (0.5) | 0.002 |
| Diagnosis | Secondary parkinsonism | 71 (0.1) | 90 (0.1) | 0.007 |
| Diagnosis | Nicotine dependence | 15,955 (15.9) | 16,056 (16.0) | 0.003 |
| Diagnosis | Tobacco use | 6,598 (6.6) | 6,641 (6.6) | 0.002 |
| Diagnosis | Personal history of nicotine dependence | 22,664 (22.6) | 23,034 (23.0) | 0.009 |
| Diagnosis | Physical debility | 103 (0.1) | 92 (0.1) | 0.004 |
| Diagnosis | Sarcopenia | 20 (0.0) | 19 (0.0) | 0.001 |
| Diagnosis | Severe protein-calorie malnutrition | 565 (0.6) | 557 (0.6) | 0.001 |
| Diagnosis | Moderate/mild protein-calorie malnutrition | 731 (0.7) | 769 (0.8) | 0.004 |
| Diagnosis | Unspecified protein-calorie malnutrition | 783 (0.8) | 772 (0.8) | 0.001 |
| Diagnosis | Abnormal weight loss | 5,163 (5.2) | 5,184 (5.2) | 0.001 |
| Diagnosis | Cachexia | 65 (0.1) | 53 (0.1) | 0.005 |
| Diagnosis | Hepatic fibrosis | 833 (0.8) | 822 (0.8) | 0.001 |
| Diagnosis | Cirrhosis | 2,573 (2.6) | 2,565 (2.6) | 0.001 |
| Diagnosis | Portal hypertension | 680 (0.7) | 666 (0.7) | 0.002 |
| Medication | Atorvastatin | 29,959 (29.9) | 30,569 (30.5) | 0.013 |
| Medication | Metformin | 21,160 (21.1) | 21,212 (21.2) | 0.001 |
| Medication | Rosuvastatin | 11,001 (11.0) | 11,136 (11.1) | 0.004 |
| Medication | Simvastatin | 12,638 (12.6) | 12,811 (12.8) | 0.005 |
| Medication | Pravastatin | 8,637 (8.6) | 8,636 (8.6) | <0.001 |
| Medication | Insulin | 21,111 (21.1) | 21,784 (21.8) | 0.016 |
| Medication | Semaglutide | 941 (0.9) | 988 (1.0) | 0.005 |
| Medication | Liraglutide | 1,534 (1.5) | 1,568 (1.6) | 0.003 |
| Medication | Dulaglutide | 1,588 (1.6) | 1,655 (1.7) | 0.005 |
| Medication | Tirzepatide | 16 (0.0) | 18 (0.0) | 0.002 |
| Medication | Empagliflozin | 2,154 (2.2) | 2,213 (2.2) | 0.004 |
| Medication | Dapagliflozin | 860 (0.9) | 874 (0.9) | 0.002 |
| Medication | Canagliflozin | 1,167 (1.2) | 1,183 (1.2) | 0.001 |
| Medication | Sertraline | 6,669 (6.7) | 6,742 (6.7) | 0.003 |
| Medication | Escitalopram | 6,601 (6.6) | 6,684 (6.7) | 0.003 |
| Medication | Fluoxetine | 3,751 (3.7) | 3,861 (3.9) | 0.006 |
| Medication | Venlafaxine | 3,979 (4.0) | 4,061 (4.1) | 0.004 |
| Medication | Duloxetine | 5,812 (5.8) | 5,988 (6.0) | 0.007 |
| Medication | Bupropion | 5,575 (5.6) | 5,640 (5.6) | 0.003 |
| Medication | Mirtazapine | 2,161 (2.2) | 2,228 (2.2) | 0.005 |
| Medication | Trazodone | 7,352 (7.3) | 7,593 (7.6) | 0.009 |
| Medication | Alprazolam | 8,472 (8.5) | 8,632 (8.6) | 0.006 |
| Medication | Lorazepam | 14,315 (14.3) | 14,680 (14.7) | 0.010 |
| Medication | Clonazepam | 4,440 (4.4) | 4,475 (4.5) | 0.002 |
| Medication | Diazepam | 8,829 (8.8) | 9,012 (9.0) | 0.006 |
| Medication | Temazepam | 2,283 (2.3) | 2,346 (2.3) | 0.004 |
| Medication | Zolpidem | 10,546 (10.5) | 10,784 (10.8) | 0.008 |
| Medication | Eszopiclone | 800 (0.8) | 858 (0.9) | 0.006 |

SMD = standardized mean difference. All 79 displayed characteristics were included in the repeat main propensity-score model. The maximum postmatching SMD was 0.016. Medication rows are recorded RxNorm ingredient concepts and do not establish active or concurrent use, dispensing, dose, duration, adherence, or indication.

**SUPPLEMENTARY FIGURE LEGENDS**

**Figure S1. Longer follow-up and simpler-adjustment sensitivity analyses for incident cognitive outcomes in non-obese versus obese adults with MASLD**

The longer follow-up analysis restricted index dates to 2016–2020 and assessed outcomes from years 1 through 10. The simpler adjustment model matched on age, sex, race/ethnicity, and tobacco use/history. The repeat main and additional analyses are reported in Tables S5–S8.

**Figure S2. Absolute event risks for cognitive outcomes in the propensity score–matched primary analysis**

Absolute risks are shown as percentages during the outcome window after excluding patients with the relevant outcome before the window.
